# Ultra-Sensitive and Rapid Detection of Pathogenic *Yersinia enterocolitica* Based on the CRISPR/Cas12a Nucleic Acid Identification Platform

**DOI:** 10.3390/foods11142160

**Published:** 2022-07-21

**Authors:** Yiran Xiao, Honglin Ren, Pan Hu, Yang Wang, Han Wang, Yansong Li, Kai Feng, Cong Wang, Qi Cao, Yuxi Guo, Zengshan Liu, Shiying Lu

**Affiliations:** 1 State Key Laboratory for Zoonotic Diseases, Key Laboratory for Zoonosis Research of the Ministry of Education, Institute of Zoonosis, College of Veterinary Medicine, Jilin University, Changchun 130062, China; xiaoyr20@mails.jlu.edu.cn (Y.X.); renhl@jlu.edu.cn (H.R.); hupan84@163.com (P.H.); ccwangy@126.com (Y.W.); wang_h19@mails.jlu.edu.cn (H.W.); l_ys92305@163.com (Y.L.); congwang20@mails.jlu.edu.cn (C.W.); cccqqqqcq@163.com (Q.C.); yxguo21@mails.jlu.edu.cn (Y.G.); zsliu1959@163.com (Z.L.); 2 Jilin Province Positioning Slaughter Management Office, Xi’an Road, Changchun 130062, China; fengfranky@163.com

**Keywords:** pathogenic *Yersinia enterocolitica*, CRISPR/Cas12a, recombinase polymerase amplification (RPA), nucleic acid, detection

## Abstract

*Yersinia enterocolitica* is a dangerous foodborne human pathogen that mainly causes gastroenteritis. Ideal methods for the detection of pathogens in food should be rapid, sensitive, specific, and cost effective. To this end, novel in vitro nucleic acid identification methods based on clustered, regularly interspaced short palindromic repeats (CRISPR)-associated protein (Cas) endonuclease have received increasing attention. In this study, a simple, visual, and ultrasensitive method, based on CRISPR/Cas12a with recombinase polymerase amplification (RPA), was developed for the detection of *Y. enterocolitica*. The results show that a specific attachment invasion locus gene (*ail*) can be rapidly detected using a CRISPR/Cas12a-RPA-based system. Application of the method to raw pork, which was artificially infected with *Y. enterocolitica*, achieved an estimated detection limit of 1.7 CFU/mL in less than 45 min, and this was 100 times lower compared with qPCR. The results indicated that the CRISPR/Cas12a-RPA system has good potential for monitoring pathogenic *Y. enterocolitica* in the chilled meat supply chain.

## 1. Introduction

*Yersinia enterocolitica* is the causative agent of yersiniosis, a zoonotic pathogen which can present in different diseases such as food poisoning, colitis, and mesenteric lymphadenitis. *Y. enterocolitica* is a Gram-negative bacterium that can proliferate at 4 °C (i.e., under refrigerated storage), and is commonly transmitted by the consumption of contaminated foods, such as raw pork [1,2].

The pathogenicity of *Y. enterocolitica* depends on many genetically stable virulence markers encoded by genes located on the 70 kb virulence plasmids and chromosomes [3]. *Y. enterocolitica* species can be classified into six biotypes: 1A, 1B, 2, 3, 4, and 5, and more than 70 different serotypes [4]. Except for biotype 1A, all biotypes are pathogenic to humans in varying degrees [5]. Biotype 1A has neither virulence plasmid *pYV* nor chromosomal virulence genes (*ail*, *ystA*), and is usually considered non-pathogenic [6]. The main virulence genes of pathogenic *Y. enterocolitica* are distributed in *ail*, *ystA*, *virF*, and *yadA*. However, *virF* and *yadA*, which are plasmid virulence genes, are easily lost during the production process [7]. The *ail* gene is an important virulence marker and is widely used in the pathogenicity analysis of *Y. enterocolitica* [6].

The presence of pathogenic *Y. enterocolitica* in foods can arise from originally contaminated raw materials (e.g., pork, unpasteurized milk) or secondary contamination during processing, and its incidence as a major cause of foodborne disease has caused widespread public concern. Pathogenic *Y. enterocolitica* can affect all populations, but children under five years of age, the elderly, and people with reduced immunity are at greater risk. Depending on the age/physical condition of the patient, the disease can present with fever, abdominal pain, and diarrhea, and often resembles appendicitis in adult patients [8,9]. The Centers for Disease Control and Prevention reported that about 117,000 people in the USA were infected with pathogenic *Y. enterocolitica*, and 35 subsequently died [1]. In the last century, China suffered an epidemic due to *Y. enterocolitica* infecting more than 500 people [10]. From June 2015 to June 2016, 1588 Chinese food samples were detected and 37 (2.33%) were contaminated with *Y. enterocolitica; Y. enterocolitica* was detected in 13 (87%) of the 15 cities surveyed in China, with prevalence rates ranging from 0 in Macau and Lhasa to 5.66% in Nanjing [11].Although the incidence of pathogenic *Y. enterocolitica* infection in the small intestine is carefully monitored in developed countries, there are no adequate diagnostic methods in Africa and the Middle East [12].

The low levels of pathogenic *Y. enterocolitica* present in the environment and food require sensitive and selective methods of detection. Consequently, most current methods are based on nucleic acid, immunology, and biosensor techniques [13]. Nucleic acid detection methods for pathogenic *Y. enterocolitica* are mainly based on PCR, multiplex PCR, qPCR, and RPA [14,15,16]. Although PCR is widely used for foodborne pathogenic bacteria [17], the associated complex (high-cost) instrumentation and laboratory requirements for visualization of the results (gel electrophoresis) with traditional PCR can prohibit its use in underdeveloped countries/regions with limited resources, and in onsite diagnostics [18]. Compared with PCR, the amplification process of qPCR is presented in real time [19], and it can provide more robust, specific, and sensitive quantitative results. Based on these characteristics of qPCR, it has been used for the detection of *Y. enterocolitica* in feces and food [20,21]. RPA is a recent isothermal nucleic acid amplification technology, and its development in molecular diagnostics is often motivated by its simplicity. Since it does not require complex instrumentation, it may be more suitable for on-site detection applications [22]. However, the relatively long amplification times and requirement for expensive instrumentation and reagents limit the practical application of these methods, particularly in resource-limited laboratories and field assays [23]. Consequently, access to sensitive, rapid, and simple assays is required to meet the current demands of pathogen detection [13].

Clustered regularly interspaced short palindromic repeats (CRISPR) is a powerful genome editing tool that has also been used in cell imaging and biosensing [24,25,26,27]. CRISPR and its associated proteins (Cas) exhibit an adaptive immune system guided by the RNA used by bacteria and archaea to defend against viral infections [28]. CRISPR/Cas12a is guided by single-stranded RNA (crRNA) and, after specific binding of target DNA, structural changes in Cas12a result in its cis- and trans-cleavage of target and non-target DNA, respectively [29,30,31]. Recombinant enzyme polymerase amplification (RPA) is a highly sensitive and selective isothermal amplification method, and its operating temperature (37–42 °C) and minimal simple preparation are of great importance for on-site detection applications [32]. Therefore, combining CRISPR/Cas12a enzymology with RPA permits the development of specific high-sensitivity enzymatic reporter unlocking probes (SHERLOCK) for nucleic acid detection. Cas12a has a lower detection sensitivity than Cas13, but when used for DNA detection, it ignores the requirement for transcriptional processes and is suitable for direct reaction after crude genome extraction. When Cas12a is combined with RPA, it has the potential for detection sensitivities at the 10^−18^ M level [33,34]. While the CRISPR/Cas12a detection platform has been applied to many foodborne pathogenic bacteria (*Escherichia coli*, *Listeria monocytogenes*, *Staphylococcus aureus*, etc.), an equivalent procedure has not been reported for *Y. enterocolitica* [35,36,37].

**Figure 1:**
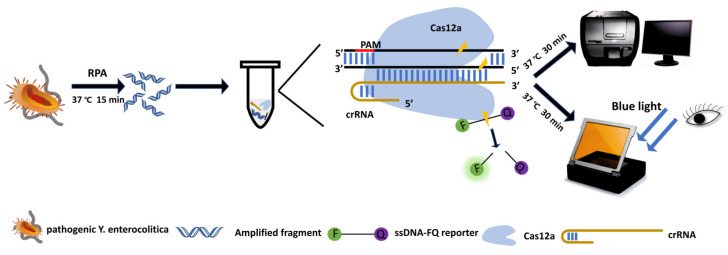
Schematic of CRISPR/Cas12a-RPA system showing extraction of the crude genomic DNA (boiling/centrifugation), RPA (37 °C, 15 min), Cas12a collateral cleavage reaction (37 °C, 30 min), and observation of the signal (blue light, or microplate reader).

Here, bacterial genome extraction, RPA, and Cas12a/crRNA cleavage analysis were integrated to develop a rapid, sensitive, and simple method for the detection of pathogenic *Y. enterocolitica* in raw pork, without reliance on laboratory facilities. The principle is based mainly on the specific recognition of target DNA by crRNA to activate the enzyme cleavage activity of Cas12a to achieve visual detection (Figure 1). This detection system may provide a basis for cost-effective and practical monitoring of *Y. enterocolitica* in the chilled meat supply chain.

## 2. Materials and Methods

### 2.1. Materials

Pathogenic *Y. enterocolitica* (CMCC52225) was obtained from the Chinese General Microbiological Culture Collection Center (CGMCC; Beijing, China); non-pathogenic *Y. enterocolitica*, *Listeria monotygenes*, *Klebsiella pneumoniae*, and *Shigella flexneri* were obtained from the Key Laboratory of Zoonoses, Ministry of Education, Jilin University (Changchun, China). Primers were synthesized by Sangon Biotech (Shanghai, China). LbCas12a, NEBuffer 3.1, single-stranded DNA (ssDNA; quenched fluorescent DNA reporter 6FAM-TTTTTT-BHQ1), and crRNA were purchased from Bio-lifesci (Guangzhou, China). The TwistAmp Basic kit was purchased from TwistDx Ltd., (Maidenhead, UK). Raw pork samples were purchased from local fresh food supermarkets.

### 2.2. Bacteria Culture and Crude Genomic DNA Extraction

The *ail* gene of pathogenic *Y. enterocolitica* was cloned into a pUC57 vector and transformed into competent *E. coli* DH5α. The copy number of pUC57-*ail* plasmid containing a 537 base-pair (bp) pathogenic *Y. enterocolitica ail* sequence was calculated on the basis of the plasmid and insert molecular weight: number of copies = (amount × 6.022 × 10^23^)/(length × 1 × 10^9^ × 650) (https://toptipbio.com/dna-copy-number-qpcr/, accessed on 12 July 2021). The pUC57-*ail* plasmid was serially diluted from 10^8^ copies/μL to 10^0^ copies/μL. *Y. enterocolitica* and negative controls, such as non-pathogenic *Y. enterocolitica* were cultured in Luria–Bertani medium at 28 °C; *Listeria monotygenes*, *Klebsiella pneumoniae*, and *Shigella flexneri* were cultured at 37 °C. Serial dilutions were made from the culture plate for the preparation of 10^8^ to 10^0^ colony-forming units (cfu/mL). To obtain crude genomic DNA for subsequent amplification, one milliliter of bacterial solution was centrifuged at 13,680× *g* for 2 min, the supernatant was removed, the bacteria were resuspended in sterile deionized water, and the centrifugation/supernatant removal was repeated. The bacteria were then resuspended in sterile deionized water (100 μL) and heated for 20 min at 100 °C, followed by centrifugation at 13,680× *g* for 2 min, and the supernatant was retained as the substrate. The purity of genomic DNA was determined from the ratio of the absorbances at 260 and 280 nm using a nanodrop spectrophotometer (Thermo Fisher Scientific, Shanghai, China).

### 2.3. Sample Preparation

Homogenized pork (1 g) was added to different concentrations of diluted bacterial solution, followed by saline solution (8 mL). After mixing, an aliquot of the matrix solution (1 mL) was subjected to DNA extraction for CRISPR/Cas12a-RPA analysis.

### 2.4. Optimization of the RPA Assay

Primers for RPA reaction were designed with Primer Premier 5.0 (PREMIER Biosoft International, San Francisco, CA, USA). The primer length was about 30 nucleotides (nt), and the expected amplicon size was under 500 bp. RPA reactions were performed according to the instructions of the Twist-Amp Basic kit (https://www.twistdx.co.uk/support/, accessed on 8 May 2021). Briefly, rehydration buffer (29.5 μL), forward and reverse primers (10 μM, 2.4 μL each), crude genomic DNA, and diethyl pyrocarbonate (DEPC) water (combined volume 13.2 μL) were mixed. Magnesium acetate (280 mM, 2.5 μL) was added to start the reaction, and the mixture was incubated at 37 °C for 15 min. The amplified products were purified using phenol/chloroform and electrophoresed on 2% agarose gel. The amplification was subsequently optimized using reaction times of 10, 15, and 20 min, temperatures of 37, 39, and 41 °C, and primer concentrations of 320, 400, and 480 nM.

### 2.5. Target Cleavage Assays Based on Cas12a

The Cas12a-mediated cleavage assay contained LbCas12a (100 nM), crRNA (100 nM), ssDNA-FQ reporter (500 nM), and NEBuffer 3.1 (2 μL) with pre-amplified products (2 μL). The total volume was adjusted to 20 μL with DEPC water. Reactions were performed in a 384-well microplate incubated in a Cytation 5 microplate reader (BioTek Instruments, Winooski, VT, USA) for 30 min at 37 °C; fluorescence measurements were recorded at 1 min intervals (λ_ex_: 485 nm; λ_em_: 528 nm). For direct visualization, the reaction tubes containing 20 μL of Cas12a reactions were placed under the blue light (470 nM) of a TGreen OSE-470L Transilluminator (Tiangen, Beijing, China); digital images were recorded using a smartphone camera.

### 2.6. Sensitivity and Specificity of CRISPR/Cas12a-RPA System

Sensitivity and specificity were evaluated after the completion of the primer and RPA reaction system optimization. Gradient dilutions of DNA, plasmid, and bacterial solutions were used for the sensitivity tests, and bacterial specificity was assessed using four *ail*-negative strains of bacteria (i.e., *Listeria monotygenes*, *Klebsiella pneumoniae*, *Shigella flexneri*, and non-pathogenic *Y. enterocolitica*). All results were analyzed using the Cas12a cleavage reaction, and the fluorescence was recorded using the blue light transilluminator and microplate reader.

### 2.7. qPCR and Standard Curves

For the comparison of CRISPR/Cas12a-RPA with qPCR, the method of [35] was followed using 2 × M5 HiPer Realtime PCR Super mix (SYBRgreen, with anti-Taq) according to the manufacturer’s instructions (Mei5bio, Beijing, China). Each reaction contained one 2× Mix (10 μL), forward and reverse primers (0.5 μL), an appropriate amount of genomic DNA, and sterile water (up to 20 μL).

### 2.8. Statistical Analysis

Experiments were performed in triplicate and data reported as means ± SD. Statistical analysis was performed using GraphPad Prism version 8.0.0 for Windows (GraphPad Software, San Diego, CA, USA). Differences in sample means were assessed using a two-tailed Student’s *t*-test; *p* < 0.05 was considered statistically significant.

## 3. Results

### 3.1. Design of Primers and crRNA

**Figure 2:**
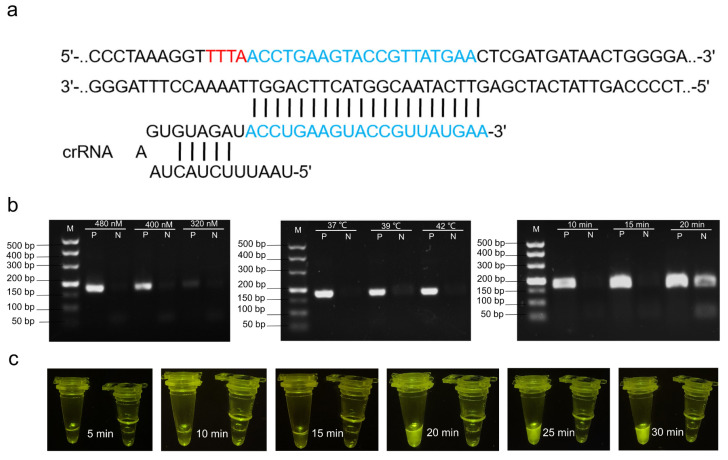
Optimization of the CRISPR/Cas12a-RPA system: (**a**) sequences of targets and crRNA designs used for the detection of pathogenic *Y. enterocolitica*; (**b**) optimization of RPA primer concentration, temperature, and reaction time (lanes N and P are negative and positive samples amplified by RPA under the different conditions; M is 500 bp marker); (**c**) visualization of the results at different time intervals.

According to previous research, *ail* was used as the target gene for pathogenic *Y. enterocolitica*, and three pairs of RPA primers targeting the marker gene were designed (Table 1). Primers *ail*-1F/R and *ail*-3F/R are used to amplify the expected fragments in PCR reactions (Figure S1a), although *ail*-1F/R is also suitable for the RPA reaction system (Figure S1b). Therefore, *ail*-1 F/R was selected as the candidate primer combination for the RPA reaction. High-quality crRNA, complementary to the DNA target, was designed using the Benchling platform (Benchling, San Francisco, CA, USA; https://www.benchling.com, accessed on 14 May 2021) (Figure 2a and Table 1).

### 3.2. Optimization of CRISPR/Cas12a Reaction System

RPA was optimized for maximum target generation. DNA was extracted via boiling, as this is considered to be the method of choice for its rapid extraction in high yields from gram-negative bacteria. The results obtained from the sequential optimization of primer concentrations (320, 400, 480 nM), amplification temperatures (37, 39, and 41 °C), and reaction times (10, 15, and 20 min) were 480 nM, 37 °C, and 15 min, respectively (Figure 2b). Excitingly, under the optimized conditions of primer concentration and time, RPA pre-amplification and CRISPR/Cas12a detection temperatures were identical, which simplified operations. The progression of the fluorescence signal with time is shown in Figure 2c.

### 3.3. Specificity of CRISPR/Cas12a System

**Figure 3:**
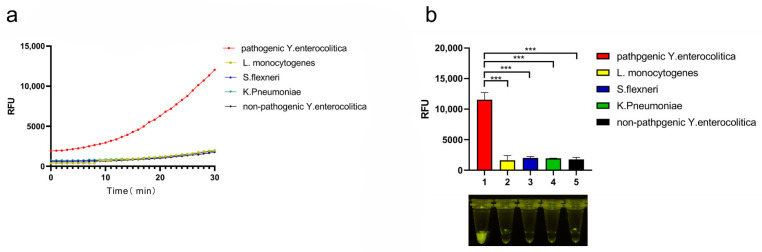
The specificity of Cas12a/crRNA direct cleavage assay: (**a**) Cas12a/crRNA assay specificity towards five different bacteria (pathogenic *Y. enterocolitica, Listeria monotygenes, Klebsiella pneumoniae*, *Shigella flexneri*, non-pathogenic *Y. enterocolitica*). Kinetics for five bacteria are shown. (**b**) Cas12a/crRNA assay sensitivity analysis. Endpoint fluorescence and visualization of targets are shown. *n* = 3 for biological replicates, * *p* < 0.05, ** *p* < 0.01, *** *p* < 0.001, and **** *p* < 0.0001, NTC—non-target control, error bars represent mean ± SD.

Based on the results, it was determined that visualization results were best after 20 min. When detecting low bacterial content, the detection time was extended to 30 min for appropriate fluorescent signal. *Y. enterocolitica* cultures were boiled (100 °C) for 20 min to release crude genomic DNA for amplification reactions, while bacterial genome extraction kits were used for the remaining bacterial strains. Figure 3a,b show that the fluorescent signal obtained for the target strain was absent from the four non-target strains, demonstrating the good specificity of the CRISPR/Cas12a system.

### 3.4. Sensitivity of CRISPR/Cas12a System

**Figure 4:**
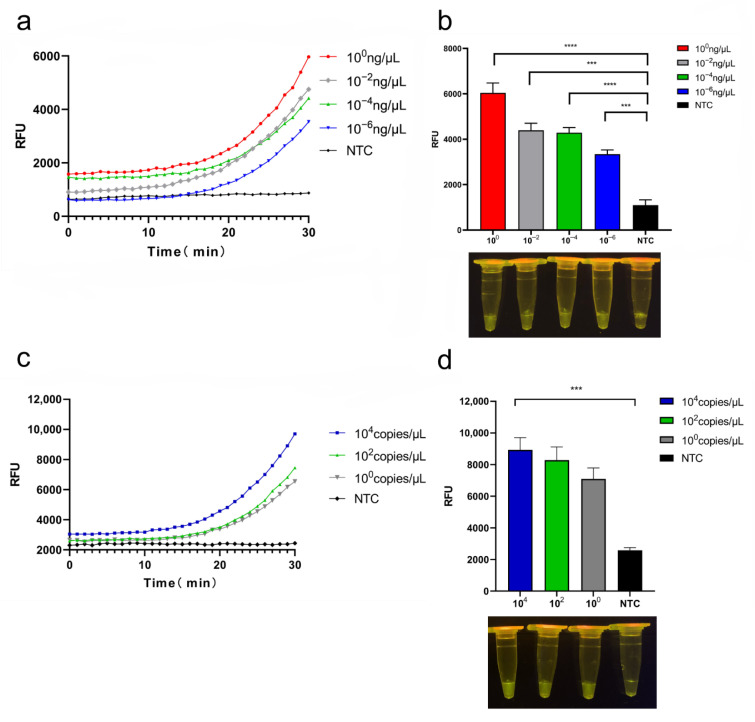
Sensitivity of the CRISPR/Cas12a system towards pathogenic *Y. enterocolitica*: (**a**) fluorescence kinetic curves for crude genomic DNA; (**b**) endpoint fluorescence and visualization of results for crude genomic assay; (**c**) fluorescence kinetic curves of the recombinant plasmid pUC57-*ail* assay; (**d**) endpoint fluorescence and visualization results of the recombinant plasmid pUC57-*ail* assay. *n* = 3 biological replicates, * *p* < 0.05, ** *p* < 0.01, *** *p* < 0.001, and **** *p* < 0.0001, NTC—non-target control; error bars represent mean ± SD.

The detection sensitivity was initially evaluated using crude genomic DNA obtained from cultures of pathogenic *Y. enterocolitica*. The results obtained from serially diluted genomic DNA (1 × 10^0^, 1 × 10^−2^, 1 × 10^−4^, 1 × 10^−6^ ng/μL) indicate a detection limit of 10^−6^ ng/μL (Figure 4a); the endpoint fluorescence and visualization of results for the crude genomic assay are shown in Figure 4b. Sensitivity was also assessed using the *ail* gene: The conserved *ail* region was cloned into the pUC57 plasmid, and DNA was serially diluted (1.25 × 10^4^ copies/μL, 1.25 × 10^2^ copies/μL, 1.25 × 10^0^ copies/μL); diluted samples (2 μL) were amplified with RPA, and the amplification product (2 μL) was used for Cas12a lysis reaction (Figure 4c); the endpoint fluorescence and visualization results of the recombinant plasmid pUC57-*ail* assay are shown in Figure 4d. The results show that the CRISPR/Cas12a system can detect the marker gene at the single-copy level, confirming the assay has high sensitivity.

**Figure 5:**
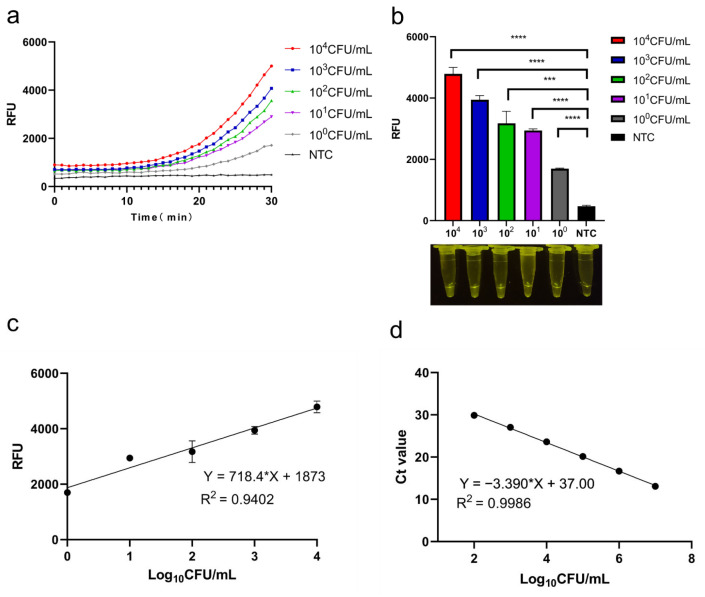
Detection of pathogenic *Y. enterocolitica* in pork matrix using the CRISPR/Cas12a-RPA system and comparison with qPCR: (**a**) CRISPR system curves from raw pork infected with different concentrations of pathogenic *Y. enterocolitica*; (**b**) endpoint fluorescence and visualization from the infected pork; (**c**) CRISPR system standard curves for the detection of pathogenic *Y. enterocolitica* in the raw pork; (**d**) standard curves for pathogenic *Y. enterocolitica* detection based on qPCR C_t_ values. *N* = 3 biological replicates, * *p* < 0.05, ** *p* < 0.01, *** *p* < 0.001, and **** *p* < 0.0001, NTC—non-target control; error bars represent mean ± SD.

The CRISPR/Cas12a system was applied to homogenized pork samples artificially contaminated with solutions of pathogenic *Y. enterocolitica* (1.7 × 10^4^, 1.7 × 10^2^, 1.7 × 10^0^ CFU/mL). The resultant kinetic curves and endpoint fluorescence exhibited robust signals (Figure 5a,b), and the detection limit was estimated to be 1.7 × 10^0^ CFU/mL. The relative fluorescence units showed a linear relationship, with concentrations of bacterial solution ranging from 10^4^ to 10^0^ CFU/mL (Figure 5c).

### 3.5. qPCR and Standard Curves

The CRISPR/Cas12a system was validated by comparing the results obtained from the artificially contaminated (pathogenic *Y. enterocolitica*) pork using quantitative qPCR. The standard curves used for the quantification of pathogenic *Y. enterocolitica* based on qPCR Ct values are shown in Figure 5d; the limit of detection was 10^2^ CFU/mL. A comparison of the two methods (in terms of resources, procedure, time, and sensitivity) is given in Table 2.

## 4. Discussion

The optimization of RPA was necessary for pre-amplification of the template, as this is related to the subsequent enzymatic cleavage activity of Cas12a. In the presence of the increased template generated by RPA, the CRISPR/Cas12a system can complete cleavage of the ssDNA probes to generate fluorescence. This combination overcomes the limitations of visualizing RPA results using (laboratory-based) gel electrophoresis.

The schematic diagram of the CRISPR/Cas12a-RPA assay is shown in Figure 1. The method was divided into four main steps: (i) extraction of the genome; (ii) RPA pre-amplification; (iii) cleavage of the target nucleic acid using Cas12a; (iiii) quantitative or visual monitoring.

The main features of the CRISPR/Cas12a-RPA system were as follows: (i) rapid RPA of the primer (480 nM, 15 min at 37 °C) and short fluorescence-intensity measurement time (30 min); (ii) screening based on the specific virulence gene *ail*, and design of RPA primers and crRNA (20 bp) for specific binding during amplification; (iii) portable detection equipment maintained at 37 °C, suitable for use in the field or in low-resource laboratories. Hence, the CRISPR/Cas12a-RPA system demonstrated good potential for the on-site monitoring of pathogenic *Y. enterocolitica* in, e.g., various food supply chains.

Despite the promising results presented here, the CRISPR/Cas12a-RPA assay has some limitations: (i) fully enclosed reaction systems need to be designed to prevent aerosol contamination. (ii) Due to the non-specific cleavage of nucleotide chains by CRISPR/Cas12a, it is challenging to perform multiplex detection in a single reaction volume. So, a microfluidic chip with micro-channels could be designed to combine and optimize reactions in a multiplexed CRISPR-based assay. Unfortunately, due to the severe impact of COVID-19 in the region, bulk samples were not available during the study for testing to verify actual infected samples.

## 5. Conclusions

A visualization method based on the CRISPR/Cas12a system was successfully developed for the detection of pathogenic *Y. enterocolitica* in food without the use of complex instrumentation. The results show that the CRISPR/Cas12a-RPA system is highly specific and extremely sensitive for pathogenic *Y. enterocolitica*. Optimization of RPA improved the yield of the template and reduced the overall detection time to 45 min. The sensitivity of the method (1.7 CFU/mL), which used pork artificially infected with pathogenic *Y. enterocolitica*, was 100 times lower compared with qPCR. In addition, the CRISPR/Cas12a-RPA assay requires only a portable thermostatic heater and a blue light projector for visualization, which facilitates on-site field testing.

## Figures and Tables

**Table 1:** Sequences of oligonucleotides used in this study.

Method	Primer’s Name	Sequences (5′-3′)	Product Length
RPA	*ail*-1F	GTCTGTTAATGTGTACGCTGCGAGTGAAAG	180 bp
	*ail*-1R	TATCCCTGATGAGTATAAGCAAACGAACCT	
	*ail*-2F	ACGCTGCGAGTGAAAGTAGT	254 bp
	*ail*-2R	TTCGTTGATGCGGAAAGATG	
	*ail*-3F	TAATGTGTACGCTGCGAGTGAAAGTAG	171 bp
	*ail*-3R	CCCTGATGAGTATAAGCAAACGAACCT	
CRISPR/Cas12a	crRNA	UAAUUUCUACUAAGUGUAGAUACCUGAAGUACCGUUAUGAA	
	ssDNA reporter	6-FAM-/TTTTTT/BHQ-1	
qPCR	F	TAATGTGTACGCTGCGAGTGAAAGTAG	
	R	CCCTGATGAGTATAAGCAAACGAACCT	

**Table 2:** Comparison between CRISPR/Cas12a-RPA and Standard qPCR.

	CRISPR/Cas12a-RPA	qPCR
Equipment	Blue light transilluminator	ABI 7500 Fast
Procedure	Easy	Moderate
Time	45 min	2 h
Sensitivity	High (10^0^ CFU/mL)	Low (10^2^ CFU/mL)

## Data Availability

The data presented in this study are available on request from the corresponding author.

## Supplementary Materials

The following supporting information can be downloaded at: https://www.mdpi.com/article/10.3390/foods11142160/s1, Figure S1: Primers screening (a) PCR amplification for *ail*-1, *ail*-2 and *ail*-3 screening; (b) RPA amplification for *ail*-1 and *ail*-3 screening.
