# Supplementary material for: Ultra-Sensitive and Rapid Detection of Pathogenic Yersinia enterocolitica Based on the CRISPR/Cas12a Nucleic Acid Identification Platform
Source: Foods. 2022 Jul 21;11(14):2160. doi: 10.3390/foods11142160 (PMC9318358; doi:10.3390/foods11142160)
Supplement: Supplementary file 1 [file foods-11-02160-s001.zip › foods-1804232-supplementary.pdf]

# Supplementary data

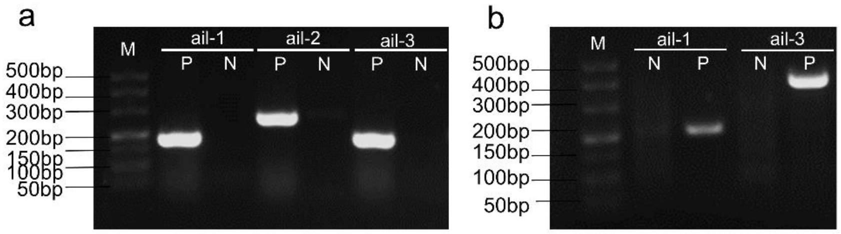


Figure S1 Primers screening (**a**) PCR amplification for *ail*-1,*ail*-2 and *ail*-3 screening; (**b**) RPA amplification for *ail*-1 and *ail*-3 screening.
